# Equine Arteritis Virus Does Not Induce Interferon Production in Equine Endothelial Cells: Identification of Nonstructural Protein 1 as a Main Interferon Antagonist

**DOI:** 10.1155/2014/420658

**Published:** 2014-05-25

**Authors:** Yun Young Go, Yanhua Li, Zhenhai Chen, Mingyuan Han, Dongwan Yoo, Ying Fang, Udeni B. R. Balasuriya

**Affiliations:** ^1^Maxwell H. Gluck Equine Research Center, Department of Veterinary Science, University of Kentucky, Lexington, KY 40546, USA; ^2^Virus Research and Testing Group, Division of Drug Discovery Research, Korea Research Institute of Chemical Technology, Daejeon 305-343, Republic of Korea; ^3^Department of Diagnostic Medicine and Pathobiology, Kansas State University, Manhattan, KS 66506, USA; ^4^Department of Infectious Diseases, University of Georgia, Athens, GA 30602, USA; ^5^Department of Pathobiology, College of Veterinary Medicine, University of Illinois at Urbana-Champaign, Urbana, IL 61802, USA

## Abstract

The objective of this study was to investigate the effect of equine arteritis virus (EAV) on type I interferon (IFN) production. Equine endothelial cells (EECs) were infected with the virulent Bucyrus strain (VBS) of EAV and expression of IFN-**β** was measured at mRNA and protein levels by quantitative real-time RT-PCR and IFN bioassay using vesicular stomatitis virus expressing the green fluorescence protein (VSV-GFP), respectively. Quantitative RT-PCR results showed that IFN-**β** mRNA levels in EECs infected with EAV VBS were not increased compared to those in mock-infected cells. Consistent with quantitative RT-PCR, Sendai virus- (SeV-) induced type I IFN production was inhibited by EAV infection. Using an IFN-**β** promoter-luciferase reporter assay, we subsequently demonstrated that EAV nsps 1, 2, and 11 had the capability to inhibit type I IFN activation. Of these three nsps, nsp1 exhibited the strongest inhibitory effect. Taken together, these data demonstrate that EAV has the ability to suppress the type I IFN production in EECs and nsp1 may play a critical role to subvert the equine innate immune response.

## 1. Introduction

Equine arteritis virus (EAV) is the causative agent of equine viral arteritis, a respiratory and reproductive disease of horses [1, 2]. EAV is a small enveloped virus with a positive-sense, single-stranded RNA genome of ~12.7 kb. It belongs to the family* Arteriviridae* (genus* Arterivirus*, order* Nidovirales*), which also includes porcine reproductive and respiratory syndrome virus (PRRSV), simian hemorrhagic fever virus (SHFV), and lactate dehydrogenase-elevating virus (LDV) of mice [3–5]. The EAV genome includes at least ten known functional open reading frames (ORFs 1a, 1b, 2a, 2b, 3, 4, 5a, 5b, 6, and 7) [5–7]. ORFs 1a and 1b are located at the 5′-proximal three-quarters of the genome and are translated to produce replicase polyproteins pp1a and pp1ab (1,727 and 3,175 amino acids, resp.). Translation of ORF1b depends on a −1 ribosomal frameshift located just before termination of ORF1a translation [8]. The two replicase precursor polyproteins are cleaved by three ORF1a-encoded proteases that reside in nsp1, nsp2, and nsp4, yielding at least 13 end-products, namely, nonstructural proteins (nsps) 1 to 12, including a recently described nsp7*α* and 7*β* [5, 9, 10]. The remaining eight ORFs (2a, 2b, and 3, 4, 5a, 5b, and 6-7) are located in the 3′ quarter of the genome and encode the structural proteins (E, GP2, GP3, GP4, ORF5a protein, GP5, M, and N, resp.) of the virus [5, 6, 11–13].

Type I interferon (IFN-*α*/*β*) is a key component of the host innate immune response to viral infection [14]. Recognition of pathogen-associated molecular patterns (PAMPs) in double-stranded RNA (dsRNA) by intracellular receptors, such as retinoic acid inducible gene I (RIG-I) and melanoma differentiation-associated antigen 5 (MDA-5) [15], activates protein signaling cascades that result in the activation of transcription factors, including interferon regulatory factor-3 (IRF-3) and nuclear factor-*κ*B (NF-*κ*B) [14]. The IFN-*β* promoter contains positive regulatory domains (PRDs), including the binding sites for different transcription factors, IRF-3 (PRDs I and III) and NF-*κ*B (PRD II). Activation of these transcription factors triggers the formation of enhanceosomes in the cell nucleus and induces the expression of IFN-*α*/*β* [14, 16]. Both IRF-3 and NF-*κ*B activation are mediated by mitochondrial antiviral signaling (MAVS) protein, which functions downstream of RIG-I and MDA-5 and upstream of the I*κ*B kinase (IKK) complex and TANK-binding kinase-1 (TBK1) [17–20]. Among the various factors involved in type I IFN production, IRF-3 plays a critical role. IRF-3 is expressed in most cell types and resides in the cytoplasm in an inactive form. When stimulated, IRF-3 becomes phosphorylated and undergoes conformational changes, resulting in dimerization with the exposure of nuclear localization signal. In the nucleus, IRF-3 recruits coactivator CBP/p300 and forms a complex to bind IRF-3 responsive elements (PRDs I and III) of the IFN-*β* promoter [14]. In addition to IRF-3, NF-*κ*B is also a critical regulator of host innate and adaptive immunity. It plays an important role in the regulation of cell proliferation as well as cell survival. Many viruses have evolved strategies to counteract key elements of the IFN response and prevent development of an antiviral response in the host [14]. Through evolution, viruses can either activate or inhibit the NF-*κ*B pathway in order to replicate in host cells. Viruses such as African swine fever virus and influenza A virus block NF-*κ*B activation to counteract the host innate immune response [21, 22]. In contrast, viruses such as hepatitis C virus, reovirus, and herpes simplex virus have developed mechanisms to directly activate NF-*κ*B to support production of progeny viruses and intracellular spreading [23–25].

Until now, the innate immune response to EAV infection was poorly characterized and the information pertaining to type I IFN production was largely derived from studies of PRRSV and other nidoviruses [26–32]. Recently, van Kasteren et al. [33] reported that the EAV PLP2 has de-ubiquitylation function which suppresses RIG-I to control innate immune signaling in EAV-infected cells [34, 35]. In all these studies, the investigators have used recombinant proteins (e.g., nsp2 protein of EAV) and a specific immune suppression mechanism. However, the effect of the whole virus and involvement of other EAV proteins in the suppression of host cell immune responses are largely unknown. To elucidate the molecular mechanism of EAV involved in host immune suppression, the objective of this study was to investigate the effect of EAV on type I IFN production and to further identify the specific viral proteins responsible for the suppression of IFN-*β* activity.

## 2. Materials and Methods

### 2.1. Virus and Cells

Equine pulmonary artery endothelial cells (EECs [36]), baby hamster kidney-21 (BHK-21 [ATCC CCL-10], Manassas, VA), and HEK293T (ATCC CRL-11228) cells were maintained in Dulbecco's modified essential medium (Mediatech, Herndon, VA) supplemented with 10% fetal bovine serum (FBS; HyClone Laboratories, Inc., Logan, UT), 100 U/*μ*g per mL penicillin-streptomycin, and 200 mM L-glutamine (Gibco, Carlsbad, CA) in a humidified incubator with 5% CO_2_ at 37°C. HeLa cells (NIH AIDS Research and Reference Reagent Program, Germantown, MD) were propagated in minimum essential medium (MEM) supplemented with 10% heat-inactivated FBS in a humidified incubator with 5% CO_2_ at 37°C. MDBK (ATCC CCL-22) cells were grown in Eagle's minimum essential medium with 10% ferritin-supplemented bovine calf serum (HyClone Laboratories, Inc., Logan, UT) and 100 U/*μ*g per mL penicillin-streptomycin (Gibco, Carlsbad, CA). The virulent Bucyrus strain of EAV (EAV VBS, horse passage 15 pleural fluid; ATCC VR-796, Manassas, VA) was passaged once in EECs to obtain high-titered working stocks for the present study using the method described previously [37]. Sendai virus, the Cantell strain (SeV; ATCC VR-907, Manassas, VA), was propagated in embryonated chicken eggs. The virus titer was determined by hemagglutination inhibition (HI) assay using chicken red blood cells as described previously [38]. Vesicular stomatitis virus expressing green fluorescent protein (VSV-GFP [39]) was kindly provided by Dr. Adolfo Garcia-Sastre (Mt. Sinai School of Medicine, New York, NY).

### 2.2. Plasmids

Plasmids for expression of recombinant EAV nsp1 to nsp12 in mammalian cells were constructed as previously described [40]. Briefly, the coding regions of each of the twelve nsps were PCR-amplified from the EAV rVBS full-length infectious cDNA clone [41] and cloned into the eukaryotic expression vector pCAGGS [33]. The nsp5, nsp6, nsp10, and nsp12 were expressed as C-terminal FLAG-tagged fusion proteins. To express the recombinant EAV nsps, BHK-21 cells were transfected with each individual plasmid containing an nsp coding region. Transfection was performed using FuGENE HD (Promega, Madison, WI) according to manufacturer's instructions. The specificity of each recombinant protein was confirmed by immunofluorescence and Western blot analysis. Reporter plasmids expressing the firefly luciferase under the control of either the IFN-*β* promoter (p125-Luc) or an artificial promoter containing three IRF-3 binding sites (p55-CIB-Luc) were kindly provided by Yoneyama et al. [42]. The pNF-*κ*B-Luc reporter plasmid (Stratagene, La Jolla, CA) expresses the firefly luciferase under the control of a promoter with NF-*κ*B-response element. The pRL-SV40 plasmid (Promega, Madison, WI) expresses a* Renilla *luciferase under the control of a simian virus (SV40) promoter. The pEFneo-RIG-I, pEFneo-MDA-5, and pEFneo-IKK*ε* were kindly provided by Komatsu et al. [43]. The pcDNA3-TRIF and pCMV2-IKK2-WT were purchased from Addgene (Cambridge, MA). Construction of the pCAGGS-IRF-3 and pCAGGS-NS1 plasmids was described previously [44].

### 2.3. Antibodies

To detect EAV antigens in infected cells, monoclonal antibodies (MAbs) against EAV nsp1 (MAb 12A4) and N protein (MAb 3E2) were used [45, 46]. Specific polyclonal rabbit antisera recognizing EAV nsp2 [47], nsp3 [48], nsp4 [47], nsp7-8 [47], and nsp10 [49] have been described previously. In addition, antisera against nsp9 and nsp11 were raised by immunizing rabbits with purified full-length recombinant proteins expressed in* E. coli* (J.C. Zevenhoven, D. D. Nedialkova, and E. J. Snijder, unpublished data). Anti-FLAG MAb (F3165) purchased from Sigma (St. Louis, MO) was used to detect FLAG-tagged EAV fusion proteins in immunofluorescence assay. Rabbit polyclonal antibodies for human IRF-3 (sc-9082) and NF-*κ*B p65 (sc-7151) were purchased from Santa Cruz Biotechnologies Inc. (Santa Cruz, CA). Alexa Fluor 488-conjugated and Alexa Fluor 594-conjugated secondary antibodies were purchased from Invitrogen (Carlsbad, CA).

### 2.4. RNA Extraction and Quantitative Real-Time RT-PCR (qRT-PCR)

Total RNA was extracted using MagMAX-96 Total RNA Isolation kit (Ambion, Austin, TX) in a MagMAX Express-96 magnetic particle processor (Applied Biosystems, Foster City, CA). RNA from each culture was treated with DNase to remove any contaminating genomic DNA (gDNA). The RNA concentration was assessed at OD_260 nm_ and purity was verified by the OD_260_/OD_280_ ratio using NanoDrop (Thermo Scientific, Wilmington, DE). The reverse transcription reaction was performed with 1 *μ*g of total RNA using RT random primers and a MultiScribe reverse transcriptase (High Capacity cDNA Reverse Transcription kit with RNase inhibitor (Applied Biosystems, Foster City, CA)) according to the manufacturer's instructions. The following IFN-*β* primers and probe set were used for PCR amplification with an Applied Biosystems 7500 Fast Real-Time PCR System: EqIL-IFN-*β*F: 5′-AATGGCCCTCCTGCTGTGT-3′, EqIL-IFN-*β*R: 5′-CCGAAGCAAGTCATAGTTCACAGA-3′, and EqIL-IFN-*β*: probe 5′-FAM-CTCCACCACGGCTC-NFQ-3′. For each sample, cDNA corresponding to the *β*-glucuronidase (*β*-GUS) gene was amplified and used as an endogenous control. All PCR efficiency values were determined using LinReg [50]. The relative concentration of target gene mRNA was equal to 2^−ΔΔC_*t*_^ where ΔΔC_*t*_ = [(Avg. gene of interest C_*t*_ − Avg.  *β*-GUS  C_*t*_)_sample_ − (Avg.  gene  of  interest  C_*t*_ − Avg.  *β*-GUS  C_*t*_)_calibrator_]. The calibrator was calculated from the mean ΔC_*t*_ of mock-infected samples for each individual gene.

### 2.5. Interferon Bioassay

The interferon bioassay was performed using a recombinant vesicular stomatitis virus (VSV) that expresses green fluorescent protein (VSV-GFP) as previously described [31, 39, 51]. Briefly, EECs were either infected with EAV or Sendai virus (SeV) alone or dually infected with both EAV and SeV at an m.o.i. of 1 and incubated for 24 h at 37°C. Culture supernatants were collected and virus in supernatant was inactivated by ultraviolet (UV) irradiation for 30 min. Two-fold dilutions of supernatants were made in DMEM and used in IFN bioassays. MDBK cells were grown in 96-well plates to 70% confluency and incubated with two-fold dilutions of each of the supernatants. After 24 h incubation at 37°C, cells were infected with VSV-GFP at an m.o.i. of 0.1 and further incubated for 18 h. Cells were fixed with 4% paraformaldehyde and expression of green fluorescence protein was examined under an inverted fluorescence microscope.

### 2.6. Cytotoxicity Test of EAV nsp1 on HEK293T Cells

HEK293T cells in 96-well plates were transfected with increased amount of plasmid expressing EAV nsp1 (0, 0.05, 0.1, 0.2, or 0.4 *μ*g/well) using FuGENE HD (Promega, Madison, WI) transfection reagent (0.8 *μ*L/well). At 24 h after transfection, cytotoxicity was determined by using a cell proliferation assay (CellTiter 96 Aqueous One Solution, Life Technologies, Grand Island, NY).

### 2.7. Luciferase Reporter Assay

HEK293T cells were seeded in 24-well plates and transfected with various combinations of plasmid DNAs: the pEFneo-RIG-I, pEFneo-MDA-5, pEFneo-IKK*ε*, pcDNA3-TRIF, or pCAGGS-IRF-3 was mixed with a plasmid expressing an EAV protein (or empty pCAGGS vector), a luciferase reporter plasmid, and the pRL-SV40 plasmid. Transfection was performed using FuGENE HD (Promega, Madison, WI) transfection reagent following the manufacturer's instruction. For the SeV or IFN stimulation, HEK293T cells were transfected with a plasmid expressing an EAV protein (or empty pCAGGS vector), a reporter plasmid, and the pRL-SV40 plasmid. The plasmid pCAGGS-NS1 expressing swine influenza virus NS1 was used as positive control. At 20 h after transfection, cells were infected with SeV at 5000 HA unit/0.5 mL/well for 12–16 h or induced by treatment with 2000 IU/0.5 mL/well of IFN-*α* or IFN-*β* for 16 h. Cells were harvested at the indicated time points. Cell lysates were subjected to reporter gene assay using the dual luciferase reporter system (Promega, Madison, WI) according to manufacturer's instruction. Firefly and* Renilla* luciferase activities were measured in a luminometer (Berthold Technologies, Oak Ridge, TN). Values for each sample were normalized using the* Renilla* luciferase values. Relative luciferase (RLU) activity is defined as the ratio of firefly luciferase reporter activity to* Renilla* luciferase activity.

### 2.8. Immunofluorescence Assay

Cells were seeded on coverslips and grown to 80% confluency. DNA transfection was performed in HeLa cells using Lipofectamine 2000 (Invitrogen; Carlsbad, CA) according to the manufacturer's instructions. A transfection mix containing DNA and Lipofectamine 2000 in OPTI-MEM I (Invitrogen; Carlsbad, CA) was incubated at room temperature for 20 min and added to each well. After incubation, the transfection mix was replaced with fresh medium, and cells were incubated for 12 h to allow gene expression. For IRF-3 staining, cells were stimulated with 1 *μ*g/mL of polyinosinic:polycytidylic [(poly(I:C)]; Sigma, St. Louis, MO) or untreated for 8 h. For NF-*κ*B p65 staining, HeLa cells were treated with 20 ng/mL of TNF-*α* for 45 min or untreated. After incubation, cells were washed with phosphate-buffered saline (PBS), fixed with 4% paraformaldehyde for 10 min at room temperature (RT), and then permeabilized using 0.1% Triton X-100 for 10 min at RT. After blocking with 1% bovine serum albumin (BSA) in PBS for 30 min, cells were incubated with primary antibody in PBS containing 1% BSA for 2 h followed by incubation with Alexa Fluor 488- and/or Alexa Fluor 594-conjugated secondary antibody for 1 h. Nuclear staining was performed with DAPI (4′,6-diamidino-2-phenylindole; Sigma, St. Louis, MO) for 3 min at RT. After washing with PBS, coverslips were mounted onto microscope slides using Fluoromount-G mounting medium (Southern Biotech, Birmingham, AL) and examined under the fluorescence microscope (Leitz Laborlux 12).

## 3. Results

### 3.1. Suppression of Type I Interferon Production by EAV

**Figure 1:**
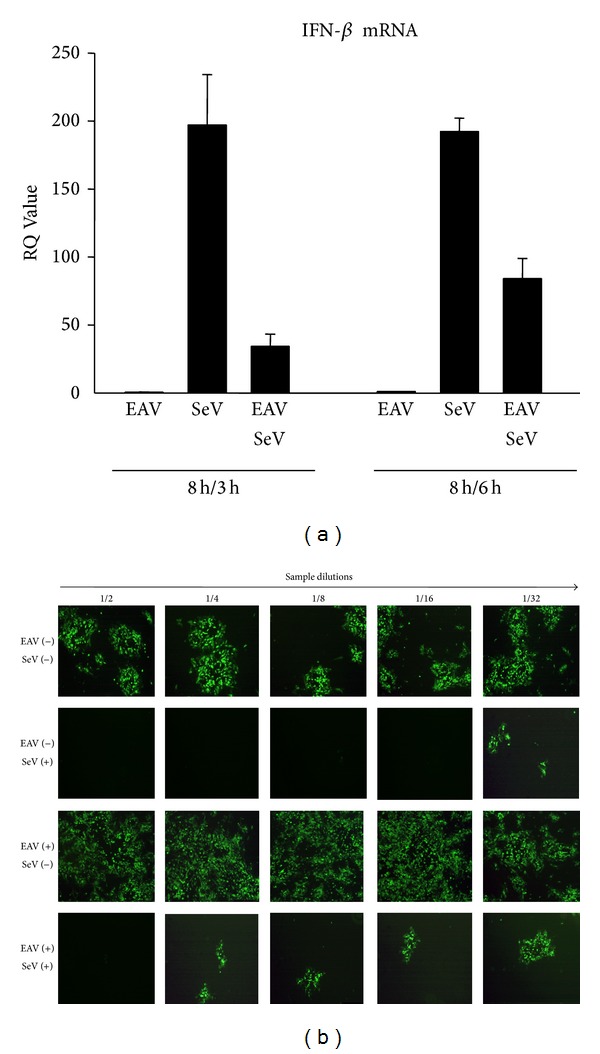
Inhibition of type I IFN production after EAV infection. (a) Expression levels of IFN-*β* mRNA in EAV infected cells. EECs were mock-infected or infected with EAV VBS at an m.o.i. of 5 for 8 h. Subsequently, cells were infected with Sendai virus (SeV; 100 HAU/mL) for 3 h or 6 h. Total RNA was isolated and real-time RT-PCR was performed for the detection of equine IFN-*β*. Bar graph showing relative quantitation (RQ) values of IFN-*β* mRNA expression from three independent experiments are shown. (b) VSV bioassay for IFN production. EECs were mock-infected or infected with EAV VBS at an m.o.i. of 1 for 24 h. SeV was used as an IFN stimulator. Cell culture supernatants were collected and UV-irradiated for 30 min prior to use in the assay. MDBK cells were grown in 96-well plates and incubated with 2-fold dilution series of the supernatant up to 1/32. After 24 h incubation, cells were infected with VSV-GFP at an m.o.i. of 0.1, and 18 h after infection GFP expression was assessed by fluorescence microscopy. Each dilution was tested in duplicate.

To investigate the effect of EAV on type I IFN production, IFN-*β* mRNA expression in infected cells was analyzed using qRT-PCR. Equine pulmonary endothelial cells (EECs) were infected with EAV VBS at an m.o.i. of 5 for 8 h, followed by infection with SeV for 3 or 6 hpi. SeV-alone-infected cells were used as positive control for type I IFN induction and mock-infected cells were used as negative control. As shown in Figure 1(a), IFN-*β* mRNA was barely detected in cells infected with EAV alone, whereas SeV infection induced a strong expression, approximately 190–200-fold increase of IFN-*β* mRNA expression level in comparison to that of mock-infected cells at 3 or 6 hpi. In contrast, SeV-induced IFN-*β* mRNA expression, at both 3 and 6 hpi, was significantly suppressed in cells previously infected with EAV. In parallel with the quantitative measurement of IFN-*β* mRNA, EAV-induced suppression of IFN production was confirmed by IFN bioassay using VSV-GFP, since VSV is IFN sensitive and presence of IFN-*α*/*β* blocks VSV replication. EECs were either infected with EAV or SeV alone or infected with both EAV and SeV. Mock-infected cells were used as negative control. Subsequently, MDBK cells were incubated with two-fold serial dilutions of virus-inactivated cell culture supernatant and then infected with VSV-GFP. The VSV infectivity was determined by monitoring the level of GFP expression. As shown in Figure 1(b), VSV-GFP replicated well in cell culture supernatant from mock-infected cells, while VSV replication was effectively inhibited in those MDBK cells that were preincubated with supernatant from SeV-infected EECs. No VSV-GFP replication was observed up to a dilution of 1 : 32 of the culture supernatant from SeV-infected cells. In contrast, VSV-GFP replication was not inhibited in MDBK cells that were preincubated with cell culture supernatant from EECs infected with EAV alone. Consistent with quantitative RT-PCR results, SeV-induced type I IFN production was significantly inhibited by EAV exposure since VSV-GFP replication was detected at a lower level compared to that from EECs only infected with SeV. Taken together, the data suggest that EAV has an ability to suppress the induction of IFN in infected cells.

### 3.2. EAV nsp1, nsp2, and nsp11 Exhibited Strong Inhibition on IFN-*β* Promoter Activation

**Figure 2:**
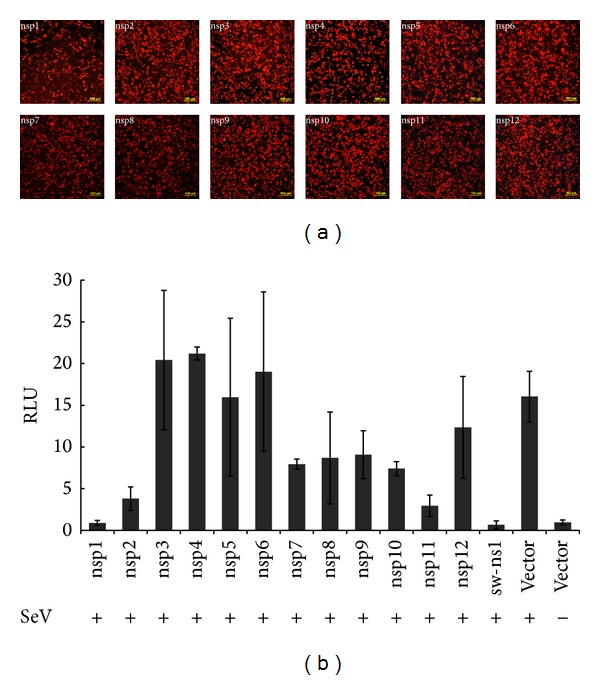
EAV nonstructural proteins (nsps) involved in suppression of IFN-promoter activation. (a) Expression of nsps in transfected cells detected by indirect immunofluorescence assay. HEK293T cells were transfected with each EAV nsp and fixed at 24 h after transfection. Cells were stained with EAV nsp1 MAb, EAV nonstructural protein-specific rabbit antisera (nsp2, nsp3, nsp4, nsp7-8, nsp9, nsp10, and nsp11), or anti-FLAG monoclonal antibody (nsp5, nsp6, and nsp12). DyLight 549-conjugated goat anti-rabbit antibody was used as secondary antibody. (b) HEK293T cells were cotransfected with p125-Luc, pRL-SV40, and pCAGGS expressing nsps or pCAGGS empty vector for 24 h. Cells were harvested and measured for firefly and* Renilla *luciferase activities. Relative luciferase (RLU) activity is defined as a ratio of firefly luciferase reporter activity to* Renilla* luciferase activity. Each data point shown represents a mean value ± standard error of the mean (SEM) from three experiments.

To investigate the role of EAV proteins as potential IFN antagonists, we focused on the nonstructural proteins of EAV. Each of the 12 nsp-encoding regions from the genome of EAV VBS was cloned individually into a mammalian expression vector, pCAGGS [40]. The expression of recombinant proteins in the plasmid DNA transfected cells was verified by immunofluorescence assay (Figure 2(a)). To determine whether these nsps have an effect on IFN-*β* activation, we used an IFN-*β* promoter-luciferase reporter assay. HEK293T cells were cotransfected with individual nsp-expressing plasmid and the luciferase reporter plasmid (p125-Luc) along with a* Renilla *luciferase expression plasmid (pRL-SV40) for normalizing purpose of sample expression levels. As a positive control, pCAGGS-NS1 plasmid expressing swine influenza virus NS1 (sw-ns1) gene was used to cotransfect the cells with the reporter plasmid, since sw-ns1 is a known IFN antagonist [52]. At 24 h after transfection, cells were infected with SeV to induce luciferase production. The IFN-*β* promoter-luciferase reporter assay result is presented in Figure 2(b). As we expected, the expression of sw-ns1 significantly inhibited IFN-*β* promoter-driven luciferase expression. In contrast, a strong reporter signal was observed in cells transfected with empty pCAGGS plasmid after infection with SeV. IFN-*β* promoter activation by SeV infection was suppressed to various degrees by expression of several EAV nsps, among which nsp1, nsp2, and nsp11 showed strong inhibition of IFN-*β* promoter-driven luciferase expression. In particular, nsp1 exhibited the strongest inhibitory effect, followed by nsp11 and nsp2. The results suggest that several EAV nsps are capable of suppressing IFN-*β* promoter activation.

### 3.3. EAV nsp1 Interferes with IRF-3- and NF-*κ*B-Mediated Signaling Pathways for IFN-*β* Production

Since the nsp1 of EAV showed the strongest inhibitory effect on the IFN-*β* promoter activation, we further determined the specific IFN production signaling pathway(s) associated with nsp1 expression. Toward this end, we tested nsp1 in IRF-3- and NF-*κ*B-promoter-driven luciferase reporter systems. Cells were cotransfected with control plasmids or with plasmids expressing the EAV nsp1 protein, the plasmid pRL-SV40, and a luciferase reporter plasmid.

**Figure 3:**
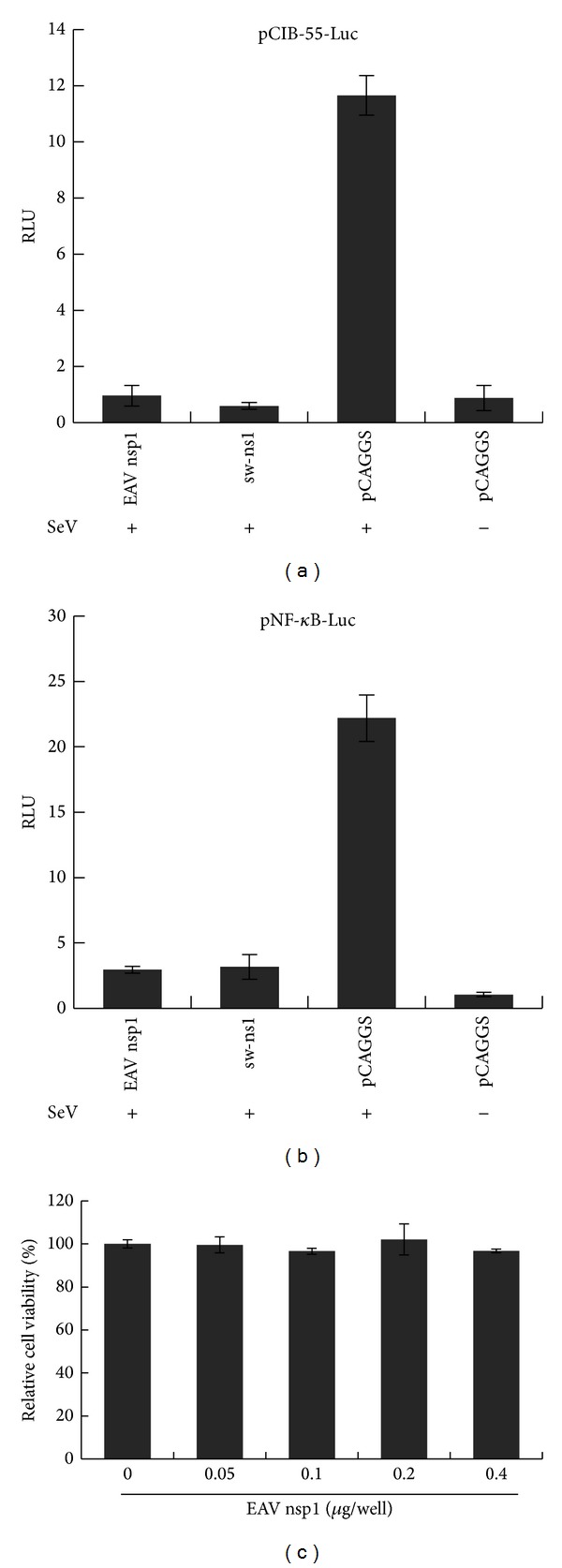
EAV nsp1 inhibits both IRF-3 and NF-*κ*B pathways. HEK293T cells were cotransfected with reporter plasmid of (a) p55-CIB-Luc and (b) NF-*κ*B-Luc and a plasmid that constitutively expresses* Renilla *luciferase and plasmids expressing EAV nsps or the indicated control plasmids. Cells were infected with Sendai virus (SeV) at 24  hours post transfection. Cells were harvested and analyzed for firefly and* Renilla* luciferase. Data were normalized using the* Renilla* luciferase values. Data are averages ± standard deviation for three experiments. (c) Cytotoxicity test of EAV nsp1 on HEK293T cells. Graph shows the results (mean and SD) of a representative quadruplicate experiment. Relative luciferase (RLU) activity is defined as a ratio of firefly luciferase reporter activity to* Renilla* luciferase activity.

As shown in Figure 3(a), upon SeV stimulation, the level of IRF-3-dependent luciferase expression was significantly reduced in cells expressing EAV nsp1 and sw-ns1 compared to that in cells transfected with control plasmid (empty pCAGGS vector). Similarly, NF-*κ*B promoter-dependent luciferase expression was suppressed in cells expressing EAV nsp1 (Figure 3(b)). These results suggest that EAV nsp1 suppresses IFN-*β* production by interfering with the IRF-3 and NF-*κ*B signaling pathways. Furthermore, the viability of the HEK293T cells expressing EAV nsp1 24 h after transfection was compared to that of untransfected control cells. As shown in Figure 3(c), the cell viability appeared to be not affected by the nsp1 protein expression. These data further suggest that the decreased type I IFN production is not due to cellular cytotoxicity, which further confirmed the important role of nsp1 in the interfering with IRF-3 and NF-*κ*B signaling pathways.

### 3.4. Effect of EAV nsp1 on the IRF-3-Dependent Signaling Pathway

**Figure 4:**
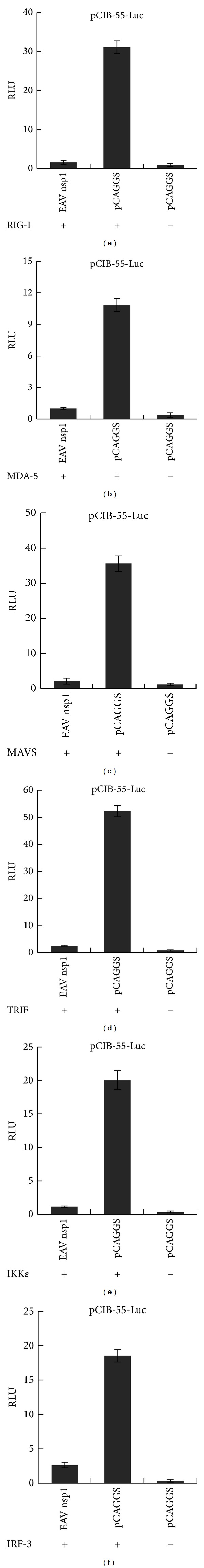
EAV nsp1 inhibits the IRF-3 signaling pathway. Suppression of (a) RIG-I, (b) MDA-5, (c) MAVS, (d) TRIF, (e) IKK*ε*, and (f) IRF-3-induced IRF-3 dependent reporter gene expression by EAV nsp1 in HEK293T cells. (a)–(f) Cells cultured in 24-well plates were cotransfected with plasmid pEFneo-RIG-I, pEFneo-MDA-5, pEGFP-N1-MAVS, pcDNA3-TRIF, pEFneo-IKK*ε*, or pCAGGS-IRF-3 along with a pCAGGS expressing nsp1 protein or pCAGGS empty vector, pRL-SV40, and a luciferase reporter plasmid p55-CIB-Luc for 24 h. Cells were harvested and measured for firefly and* Renilla* luciferase activities. Relative luciferase (RLU) activity is defined as a ratio of firefly luciferase reporter activity to* Renilla* luciferase activity. Mean value ± SEM from three experiments is shown.

We further investigated specific steps in the IRF-3 signaling pathway that EAV nsp1 could possibly block. We tested each step in the signaling pathway of IRF-3 activation. First, we investigated whether the nsp1 was interfering with the mitochondrial antiviral signaling (MAVS) complex activity. Since MDA-5 or RIG-I is associated with the MAVS complex, cells were cotransfected with a plasmid expressing RIG-I, MDA-5, or MAVS protein, a plasmid expressing EAV nsp1, the plasmid pRL-SV40, and the p55-CIB-Luc reporter plasmid. As shown in Figures 4(a)–4(c), IRF-3 promoter-dependent luciferase expression was suppressed in the presence of EAV nsp1. These results suggested that EAV nsp1 might inhibit the MAVS-mediated IFN-*β* induction or downstream portion of the signaling pathway. Therefore, we further tested the effect of EAV nsp1 on TRIF- and IKK*ε*-mediated IFN-*β* induction. The results showed that nsp1 had the ability to suppress TBK1- and IKK*ε*-mediated reporter gene expression (Figures 4(d) and 4(e)). Similarly, overexpression of IRF-3 itself did not activate the transcription of the luciferase reporter gene either (Figure 4(f)). These results suggest that EAV nsp1 might block the signaling process downstream IRF-3 activation, possibly in the nucleus.

### 3.5. Effect of EAV nsp1 on the NF-*κ*B -Dependent Signaling Pathway

**Figure 5:**
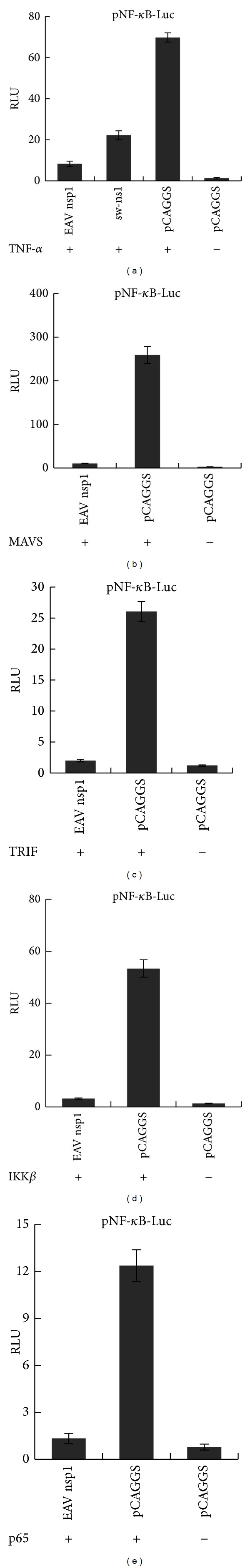
EAV nsp1 inhibits the NF-*κ*B signaling pathway. Suppression of (a) TNF-*α*, (b) MAVS, (c) TRIF, (d) IKK*β*, and (e) p65 subunit of NF-*κ*B-induced NF-*κ*B-dependent reporter gene expression by EAV nsp1 in HEK293T cells. (a) Cells cultured in 24-well plates were cotransfected with pNF-*κ*B-Luc, pRL-SV40, and pCAGGS expressing nsp1 or pCAGGS empty vector for 24 h and subsequently stimulated with TNF-*α* (20 ng/mL) for 6 h. (b)–(e) Cells were cotransfected with plasmid pEGFP-N1-MAVS, pcDNA3-TRIF, pCMV2-IKK2-WT, or pEGFP-N1-p65 along with a pCAGGS expressing nsp1 protein or pCAGGS empty vector, pRL-SV40, and a luciferase reporter plasmid pNF-*κ*B-Luc for 24 h. Cells were harvested and measured for firefly and* Renilla* luciferase activities. Relative luciferase (RLU) activity is defined as a ratio of firefly luciferase reporter activity to* Renilla* luciferase activity. Mean value ± SEM from three experiments is shown.

Since EAV nsp1 also inhibited the activation of the NF-*κ*B-dependent signaling pathway (Figure 3), the mechanism by which EAV nsp1 can inhibit the NF-*κ*B signaling pathway was analyzed in detail. As shown in Figure 5(a), in the presence of EAV nsp1, NF-*κ*B-dependent reporter gene expression was strongly inhibited with the stimulation of the TNF-*α*, a potent inducer for the activation of NF-*κ*B signaling pathway. Subsequently, the effect of EAV nsp1 on the MAVS complex and Toll/interleukin-1 receptor domain-containing adaptor protein (TRIF-) and IkB kinase beta (IKK*β*-) mediated NF-*κ*B activation was evaluated. Overexpression of any of these proteins induced activation of NF-*κ*B-dependent reporter gene expression. However, when cells coexpressed the EAV nsp1 with one of these signaling molecules, the expression level of luciferase reporter was significantly reduced (Figures 5(b)–5(d)). Similarly, overexpression of p65, a subunit of the NF-*κ*B complex, had no effect on the activation of the transcription of NF-*κ*B-driven reporter gene, but EAV nsp1 significantly reduced the expression level of luciferase reporter signal (Figure 5(e)).

### 3.6. Effect of EAV nsp1 Expression on Nuclear Translocation of IRF-3 and NF-*κ*B

**Figure 6:**
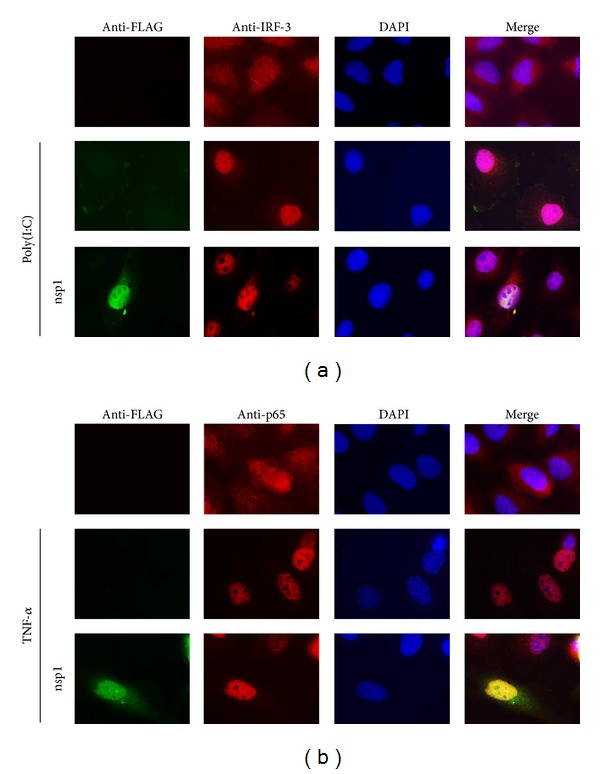
Effect of EAV nsp1 in IRF-3 and NF-*κ*B nuclear translocation. (a) HeLa cells were transfected with FLAG-tagged EAV nsp1 for 12 h and cells were stimulated with poly(I:C) (1 *μ*g/mL) for 8 h. Cells were fixed and stained with rabbit anti-IRF-3 Ab for endogenous IRF-3 and mouse anti-FLAG Ab for FLAG-tagged EAV nsp1, followed by incubation with Alexa Fluor 594-conjugated (red) and 488-conjugated (green) secondary antibodies, respectively, along with DAPI for nucleus staining (blue). (b) HeLa cells were transfected with FLAG-tagged EAV nsp1 for 12 h and subsequently treated with 20 ng/mL of TNF-*α* for 45 min or untreated. Cells were then costained with mouse anti-FLAG MAb for FLAG-tagged EAV nsp1 and rabbit anti p65 polyclonal antibody for endogenous NF-*κ*B p65 subunit followed by Alexa Fluor 488-labeled (green) anti-mouse Ab and Alexa Fluor 594-labeled (red) anti-rabbit Ab, respectively, along with DAPI for nucleus staining (blue).

The mechanism of EAV nsp1 effect on IRF-3- and NF-*κ*B-dependent gene expression was further examined by observing the nuclear localization of IRF-3 and NF-*κ*B, respectively. EAV nsp1 transfected HeLa cells were stimulated with poly(I:C), and subsequently expression of IRF-3 was stained with anti-IRF-3 antibody. In the absence of stimulation, IRF-3 was homogeneously distributed throughout the cell, whereas upon poly(I:C) stimulation IRF-3 was mainly translocated into the nucleus. As shown in Figure 6(a), expression of EAV nsp1 did not block the nuclear translocation of IRF-3. The result suggested that nsp1-mediated suppression of IFN production occurs downstream of the IRF-3 nuclear translocation. Similarly, the nuclear translocation of NF-*κ*B p65 subunit was determined in cells expressing EAV nsp1 by immunofluorescence assay (Figure 6(b)). NF-*κ*B remained largely not only in the cytoplasm but also in the nucleus to some extent in unstimulated cells, whereas TNF-*α* stimulation induced the nuclear translocation of p65. In cells expressing EAV nsp1, TNF-*α* stimulation did not notably change the p65 nuclear translocation, and p65 normally remained in the nucleus, indicating that EAV nsp1 did not block p65 nuclear translocation. These results suggest that, in the NF-*κβ*-dependent signaling pathway for IFN-*β* production, the activation of IFN-*β* transcription would be blocked by nsp1 somewhere downstream in the nucleus after NF-*κ*B nuclear translocation occurred.

## 4. Discussion

Synthesis and secretion of type I IFNs, such as IFN-*α* and IFN-*β*, are critical aspects of the antiviral immune response [14, 53, 54]. Viruses use different mechanisms to inhibit interferon response in order to evade the host innate immune response. Many viruses encode more than one protein capable of inhibiting the interferon response, which act synergistically to ensure complete blocking of interferon activity. For example, Ebola, Nipah, and SARS-CoV encode multiple viral proteins capable of inhibiting interferon activity, suggesting important roles for these proteins in pathogenesis and disease outcome [55–58]. In this study, we investigated whether EAV has the ability to interfere with the host innate immune response, in particular, type I IFN production. Our results demonstrated that EAV infection in EECs significantly inhibited type I IFN production at both mRNA and protein levels, whereas infection with SeV stimulated a high level of type I IFN production. Furthermore, EAV infection significantly inhibited SeV-induced type I IFN production as well. Based on IFN-*β* promoter-luciferase reporter assay results, three EAV nonstructural proteins, nsp1, nsp2, and nsp11, were identified as potential IFN antagonists. Previous studies reported that EAV nsp2-encoded papain-like proteinase (PLP2) is capable of inhibiting Ub- and ISG15-dependent innate immune responses [34, 35, 59]. The EAV nsp11 encodes NendoU endoribonuclease [60], which is highly cytotoxic upon its* in vitro* expression. In PRRSV, the homologous nsp11 was proposed to be an IFN antagonist [26, 31, 61]. However, whether this effect is due to the cytosolic version of the enzyme targeting on the overall RNA population of the cell or the specific suppression of IFN production needs to be determined in the future. It could be possible that the three potential immune antagonists of EAV, nsp1, nsp2, and nsp11, target different parts of the host cellular immune system and their synergistic effect during the course of infection could be able to shut down the host cell innate immune response completely. This may explain why the induction of interferon and some immunomodulatory cytokines are inhibited during* in vivo* EAV infection [62].

Among the three potential IFN antagonists of EAV, expression of nsp1 had the strongest inhibitory effect on IFN-*β* promoter activation. In comparison to our findings, previous studies on PRRSV also demonstrated that the EAV nsp1 homologous, PRRSV nsp1*α*/*β* has the strongest ability to inhibit type I IFN synthesis [26, 31, 51, 63–67]. Further analysis revealed that EAV nsp1 inhibited two key signaling pathways for IFN-*β* activation, the IRF-3- and NF-*κ*B-dependent signaling pathways. In our study, EAV nsp1 blocked each signaling step upstream of IRF-3 or NF-*κ*B activation, suggesting that EAV nsp1 acts downstream of all those tested steps in both signaling pathways. Immunofluorescence microscopy analysis further showed that nsp1 did not have much effect on the nuclear accumulation of IRF-3 and NF-*κ*B. Therefore, we postulated that EAV nsp1 might have an effect on the IFN-*β* promoter inside the nucleus. Previous studies have shown that PRRSV nsp1*α*/*β* modulates type I IFN response by blocking dsRNA-induced IRF-3 activation and IFN promoter activities, but IRF-3 phosphorylation and its nuclear translocation occur normally in the presence of the nsp1*α*/*β* [51]. Recent study reported that PRRSV nsp1*α* blocks the IRF-3 activation by degrading the CREB (cyclic AMP response element binding)-binding protein (CBP) and subsequently inhibiting formation of enhanceosomes in the nucleus [51, 63]. EAV nsp1 was reported to be largely nuclear located, similar to PRRSV nsp1*β* [68], suggesting that it may have an effect on the formation of the transcription enhanceosome on the IFN-*β* promoter inside the nucleus. However, PRRSV nsp1*β* does not degrade CBP or interrupt the formation of enhanceosome [68]. Thus, EAV nsp1 may have a unique function in modulating the IFN production in the nucleus. The EAV nsp1 does not contain the traditional nuclear localization signal [68], which suggests that nsp1 might be bound with cellular protein(s) and shuttled into the nucleus. Further studies are required to map the exact point(s) on the IFN induction pathway at which EAV nsp1 acts. It will be interesting to determine the requirement for nuclear localization of the EAV nsp1 that relates to its interferon antagonist function.

In summary, this study is the first report on molecular mechanisms of EAV involved in IFN antagonistic activity in its natural host cells. Our data indicate that several EAV replicase proteins, including nsp1, nsp2, and nsp11, possess IFN antagonistic activity and may have potential roles in the regulation of host innate immune responses. Among these proteins, nsp1 may play a key role as IFN antagonist. Further studies are needed to elucidate the detailed mechanisms that EAV proteins are involved in counteracting the host innate immune response.
